# Gingival overgrowth during orthodontic treatment and its management

**DOI:** 10.11604/pamj.2022.42.305.36600

**Published:** 2022-08-24

**Authors:** Aishwarya Deepaksingh Rathod, Priyanka Jaiswal

**Affiliations:** 1 Sharad Pawar Dental College and Hospital, Datta Meghe Institute of Medical Sciences (Deemed to be University), Wardha, India

**Keywords:** Gingivectomy, gingival enlargement, orthodontic

## Image in medicine

An increase in plaque retention and ineffective dental hygiene practices lead to gingival enlargement (GE). A 24-year-old female came to the department of periodontics with chief complaint of proclined teeth in the front region of the jaw, and accordingly planned for orthodontic treatment. After 10 months of orthodontic therapy, it was observed that there was GE in the upper anterior region of the teeth. Following comprehensive scaling and dental hygiene instructions, the patient was recalled three weeks later for another evaluation and a gingivectomy procedure was scheduled. The administration of local anaesthetic was carried out under all septic safeguards and circumstances. Pocket markers were used to identify bleeding spots. Then, using a no. 15 BP blade or Kirkland knife, external bevel incisions were made beyond the markings. As the lesion extended interproximally, the Orban knife was then utilised interdentally. Using a curette and scissors, tissue tags were taken off. By applying pressure packs with wet gauze or cotton infused with local anaesthetic, the bleeding was managed. Following the establishment of hemostasis, gingivoplasty with scalpel involving the thinning of the attached gingiva, tapering of the gingival edge, and shaping of the interdental papilla was carried out. Periodontal dressing was applied when the bleeding was stopped. Analgesics and an antiseptic mouthwash were prescribed for the patient as part of the postoperative instructions.

**Figure 1:**
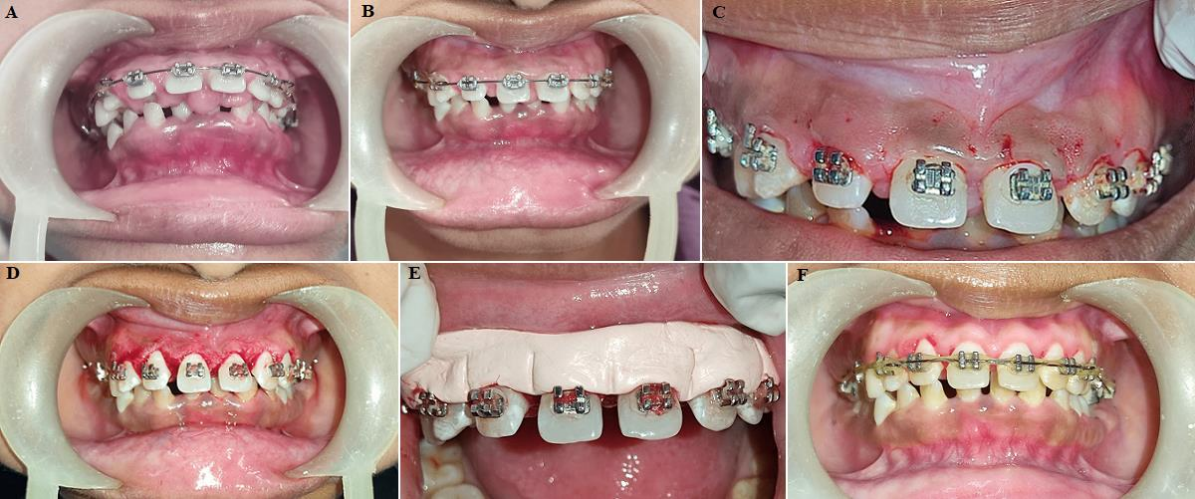
A) pre-operative; B) after scaling; C) bleeding points marked; D) immediately after treatment; E) periodontal pack; F) after 1 month of follow up

